# The health-related experiences of detained immigrants with and without mental illness

**DOI:** 10.1016/j.jmh.2025.100302

**Published:** 2025-01-04

**Authors:** Caitlin Patler, Altaf Saadi, Paola Langer

**Affiliations:** a Goldman School of Public Policy, University of California Berkeley, 2607 Hearst Ave, Berkeley, CA 94720, United States; b Department of Neurology, Massachusetts General Hospital, Harvard Medical School, Boston, MA, United States

**Keywords:** US immigration detention, Immigrants, Refugees, Mental illness, Health disparities, Healthcare interruptions, Solitary confinement

## Abstract

**Background:**

Incarcerated individuals with mental illness face unique health challenges, yet we know little about individuals with mental illness who are detained by US immigration authorities. We aimed to describe the health-related experiences of detained immigrants with and without mental illness.

**Methods:**

We conducted a cross-sectional telephone survey in 2021 with a sample of recently detained immigrants who were detained by Immigrations and Customs Enforcement (ICE) and released in the United States in 2020–2021 (*n* = 203). We used multivariable regression to assess the association between mental illness and the incidence of five outcomes while in immigration detention: 1) poor general health, 2) difficulty accessing medical services, 3) difficulty accessing mental health services, 4) interruptions to care, 5) and exposure to solitary confinement.

**Results:**

115/203 participants (56.7 %) had diagnosed mental illness, most commonly depression and PTSD. Rates of poor health, difficulty accessing medical and mental health services, interruptions to care, and exposure to solitary confinement during detention were high overall, and significantly higher among individuals with mental illness.

**Conclusion:**

There is pressing need for policy actions and protections to mitigate health harms experienced in immigration detention.

## Highlights

• We analyze health-related experiences of detained immigrants, by mental illness status.
• We find high rates of poor health, care interruptions, solitary confinement.
• Especially poor outcomes among those with mental illness.
• Immigration detention likely harmful for all detainees, particularly those with mental illness.

## Introduction

The United States maintains the world's most extensive immigration detention system. Between 2008 and 2018, U.S. Immigration and Customs Enforcement (ICE) apprehended over two million noncitizens in the US interior (non-border regions) (Transactional Records Access Clearinghouse 2018). The average daily detained population reached a historic high of 55,654 in August 2019, fell dramatically during the COVID-19 pandemic, but has continued to climb since 2021, reaching nearly 40,000 as of March 2024 (Transactional Records Access Clearinghouse 2024; Patler et al., 2023). ICE detains immigrants on civil immigration violations in over 200 jails and privately-operated facilities. Many immigrants are held mandatorily and indefinitely, with limited access to attorneys and other protections available under US criminal law.

Despite the vast scope of the immigration detention system, data on detained immigrants’ health is limited (Transactional Records Access Clearinghouse 2022). There is a particular lack of research on the experiences of detained immigrants with mental illness (MI), despite the distinct vulnerabilities this population faces in carceral settings. Emerging evidence suggests that immigration detention is detrimental to health due to mechanisms including poor conditions of confinement, substandard care, and exposure to punitive or abusive practices, all of which may be particularly harmful for individuals with mental illness (Bakely et al., 2023; Lopez et al., 2021; Nwadiuko et al., 2022; Patler and Saadi, 2021; Saadi et al., 2020; Diaz et al., 2023; Saadi et al., 2022), potentially leading to worse health outcomes.

The goal of the present study was to describe the health-related detention experiences of detained immigrants with and without mental illness. We analyze data from one of the only and most recent existing health surveys of detained immigrants in the United States. We examine the association between diagnosed MI and five outcomes while in immigration detention: 1) poor general health, 2) difficulty accessing medical services, 3) difficulty accessing mental health services, 4) interruptions in care, and 5) experience of solitary confinement. There was a high prevalence of each of these outcomes among all study participants, but especially among those with diagnosed mental illnesses, who comprise 56.7 % of our sample. Our results highlight the vulnerability of all individuals in immigration detention, particularly those with mental illness, and underscore the pressing need for policy actions and protections to mitigate harm.

## Theory & background

ICE does not release detailed health information about the populations it detains; there is therefore limited research on the experiences of individuals with MI who are detained in the US immigration detention system. This dearth of knowledge is troubling, given the distinct vulnerabilities faced by people with mental illness in carceral settings. People with MI are overrepresented in jails and prisons around the globe (Fazel et al., 2016; Fazel and Seewald, 2012; Prins, 2014) and over half of incarcerated people in the United States have at least one mental health problem (James and Glaze, 2016). Jails and prisons can also be particularly harmful for those with MI, who can experience poorer overall health, greater barriers to healthcare, and more punitive experiences within carceral settings (Canada et al., 2022; Clark, 2018; Reingle Gonzalez and Connell, 2014; Vogel et al., 2021). Mental health conditions are also risk factors for suicide and self-harm, violence, and victimization among prisoners (Fazel et al., 2016).

While the experiences of individuals with MI in jails and prisons (i.e. under criminal law) are better documented, we know far less about detained immigrants with MI. This is largely due to lack of data transparency and accessibility, despite calls for attention to the distinct needs of this population from civil and human rights organizations (Disability Rights California 2019; National Immigrant Justice Center 2022; American Immigration Council 2024). The few existing studies of detained immigrants in the United States and elsewhere suggest a high prevalence of chronic health conditions, including MI, that may initiate and/or go untreated within immigration detention facilities (Bakely et al., 2023; Patler and Saadi, 2021; Diaz et al., 2023; Keller et al., 2003; von Werthern et al., 2018). One study of 529 detained immigrants found that 43 % had at least one chronic health condition, 16 % had multiple chronic conditions, and 21 % experienced interruptions to care while in detention (Patler and Saadi, 2021).

While ICE does not release any estimates of the incidence or prevalence of MI among detained immigrants in the United States, surveys of detained or formerly detained immigrants find that nearly one in every five-to-six detained people have a diagnosed MI or neuropsychiatric disorder; however, this is likely an undercount given barriers to care (and, therefore, diagnoses) prior to and during detention (Bakely et al., 2023; Patler and Saadi, 2021; von Werthern et al., 2018). Existing research also suggests that incarcerated individuals with MI, including immigrants detained by ICE, disproportionately experience punitive conditions such as solitary confinement, despite its unique harms to their health (Franco et al., 2022; Haney, 2018; Patler et al., 2018; Reiter et al., 2020).

Mental illness and corresponding health-related outcomes in immigration detention can be caused and/or exacerbated by multiple mechanisms prior to and during detention. Many detained immigrants have experienced extensive stress and trauma in their countries of origin before migrating to the United States, as well as during the process of migration and settlement, that make them vulnerable to the health harms of a carceral setting (Saadi et al., 2020). During detention, detained immigrants are exposed to subpar and often delayed medical care, as well as to conditions of confinement that are detrimental to health including lack of nutrition, exposure to extreme temperatures, sleep deprivation, isolation from family, physical and psychological threats to safety, and punitive practices such as exposure to solitary confinement (Saadi et al., 2022; Venters and Keller, 2009; Dekker et al., 2024). As a result, detained migrants may experience high rates of MI, and those with MI may be particularly vulnerable to the health harms of immigration detention.

## Methods

### Design and data

We analyzed telephone survey data from a referral sample of 203 adult individuals released from immigration detention in 2020–2021. The survey was part of a larger sequential, longitudinal, mixed-methods, and multi-perspective study. The American Civil Liberties Union (ACLU) litigated release of detained immigrants during the COVID-19 pandemic (immigration laws otherwise provide no systematic mechanism for release) and had contact information for 355 individuals (Bansal, 2021; Plevin and R, 2020).^1^ At least 15 attempts were made to contact each individual, with the following results: 221 (62 %) completed full surveys, 11 (5 %) completed partial surveys, 21 (6 %) declined to participate, 52 (15 %) could not be reached, and 44 (12 %) were ineligible due to language barriers (*n* = 36), re-incarceration (*n* = 3), and deportation or return to country of origin (*n* = 5). The final analytical sample included 203/221 individuals (92 % of the full sample) who, after listwise deletion, had no missing responses on the variables included in our models (missingness ranged from 0 to 5.9 % [0–12 cases] and did not vary systematically). We do not have information about individuals who did not complete the survey and therefore cannot assess how closely they resemble study participants. Further, because ICE does not release detailed information about the population it detains, we cannot assess how well this study represents the entire detained population. Instead, the study provides a descriptive analysis of recently detained immigrants’ health experiences, one of only very few studies of this population.

We adapted survey materials from studies of people imprisoned/detained in the criminal and immigration legal systems, customized for a detailed focus on health (Patler and Saadi, 2021; Saadi et al., 2022; Patler and Branic, 2017; Patler et al., 2021; Western et al., 2017). Our 93-question survey gathered data about health and access to care prior to, during, and after detention; demographic background; work and legal history; family and household; and detention and post-release experiences. 159/203 of participants (78 %) were in California; the remainder were in 22 other US states (in post-hoc analysis, substantive results did not change when we controlled for California vs. other locations). Trained interviewers at the Social Science Research Center at California State University, Fullerton, conducted surveys in English or Spanish (median length: 45 min) a median of 11 months following release (IQR: 8.2–12.6 months). Participants received a $10 gift card for participation, as well as entry into a raffle for an iPad. All study materials and procedures were approved by the Institutional Review Boards of the University of California, Davis, Massachusetts General Hospital, and California State University, Fullerton. Analysis of deidentified survey data was determined exempt by the IRB of the University of California, Berkeley.

### Outcome measures

#### Poor health

To assess overall health during detention, participants described their health during detention on a scale of excellent, very good, good, fair, or poor. We created a binary measure of poor/fair (hereafter “poor”) health (poor/fair health=1, good/very good/excellent health=0).

#### Difficulty accessing medical services

Participants reported whether they had difficulty accessing medical services while detained (1=yes, 0=no/did not try). Substantive results do not change when we exclude “did not try” responses (*n* = 2).

#### Difficulty accessing mental health services

Participants reported whether they had difficulty accessing mental health services while detained (1=yes, 0=no/did not try). Substantive results do not change when we exclude “did not try” responses (*n* = 20).

#### Interruptions in healthcare

Participants provided information about lifetime history of diagnosed health conditions, including various MIs (we detail this question below). Participants then reported whether their medical care for any of the condition(s) was interrupted during detention (1=yes, 0=no/don't know). Substantive results do not change when we exclude “don't know” responses (*n* = 3).

#### Solitary confinement

Participants reported whether they were ever subject to solitary confinement while in detention (1=yes, 0=no/don't know) (Franco et al., 2022; Haney, 2018; Reiter et al., 2020). Substantive results do not change when we exclude “don't know” responses (*n* = 8).

### Diagnosed mental illness

We assessed diagnosed MI based on the question, “Have you ever been told by a doctor or other health care professional that you had…” that then listed 19 discrete conditions (as well as an “other” option), including the following mental health conditions: depression, post-traumatic stress disorder (PTSD), schizophrenia or bipolar disorder, or other mental health problem or condition (1=yes to any mental health conditions; 0=no/don't know to all). Substantive results do not change when we exclude “don't know” responses (*n* = 2).

### Covariates

Our regression models control for demographic, legal, and pre-detention health status measures that could influence health outcomes while in detention. We include age (in years), self-reported gender (male=1, female=0), education (1=high-school degree or more, 0=does not have a high school degree), self-reported ethnicity (1=Latina/o/Hispanic, 0=not Latina/o/Hispanic), criminal record background (1=has record, 0=no record); and length of detention (1 = 0 - <6 months, 2 = 6- <12 months, 3 = 12+ months). To proxy pre-detention health status, we also control for self-rated health in the year prior to detention (poor/fair =1; good/very good/excellent=0), health insurance access in the year prior to detention (1=had insurance, 0=did not have insurance), and the presence of any other diagnosed medical condition, excluding mental illness (1=yes, 0=no) .

### Analysis

We used logistic regression with robust standard errors to model each outcome, first conducting an unadjusted model wherein the only predictor variable was diagnosed mental illness, and second, using multivariable analysis to adjust for MI along with all other covariates. We then calculated the conditional average probability of experiencing each outcome, based on our fully adjusted models, testing whether differences between individuals with MI and without MI were statistically different from zero. To test the robustness of multiple-testing issues and Type 1 error, we implemented the Bonferroni correction and find that p-values from multiple regression analysis are below the Bonferroni-corrected p-value (0.05/5 = 0.01). We conducted all analyses using Stata 18.0.

## Results

### Participants and sample characteristics

Table 1 presents unadjusted statistics for the study sample, and by MI status, with the final column presenting results of two-tailed tests of means between participants with and without MI. The mean age was 40.3 years old (SD: 10.0), with 178 (87.7 %) identifying as male, 149 (73.4 %) as Latina/o/Hispanic, and 124 (61.1 %) having a high school degree or higher. 145 (71.4 %) had a criminal record and 55 (27.1 %) were detained for 12 months or longer. Eighty-two (40.4 %) reported poor health prior to detention and 98 (48.3 %) had health insurance prior to detention.

**Table 1:** Sample characteristics, for all and by mental illness status.

	*All* N *=203* *n (%)*		*Has MI* N *=115 n (%)*		*No MI* N *=88* *n (%)*		*Difference between MI/no MI* (p-value)*
*Outcome Measures*							
Poor Health	145	(71.4)	97	(84.3)	48	(54.5)	<0.001
Difficulty accessing medical services	116	(57.1)	80	(69.6)	36	(40.9)	<0.001
Difficulty accessing mental health services	84	(41.4)	63	(54.8)	21	(23.9)	<0.001
Care interrupted	74	(36.5)	60	(52.2)	14	(15.9)	<0.001
Experienced solitary confinement	92	(45.3)	64	(55.7)	28	(31.8)	0.001
*Mental illness status*							
Has diagnosed mental illness	115	(56.7)	115	(100)	0	(0)	–
*Demographic, legal, and pre-detention health background*							
Age (years) mean (SD)	203	40.3 (10.0)	115	40.3 (9.5)	88	40.2 (10.6)	0.897
Male (Ref.: Female)	178	(87.7)	97	(84.3)	81	(92)	0.118
Hispanic/Latina/o	149	(73.4)	81	(70.4)	68	(77.3)	0.17
High School Degree or more (Ref.: Less than HS)	124	(61.1)	72	(62.6)	52	(59.1)	0.237
Has a criminal record	145	(71.4)	81	(70.4)	64	(72.7)	0.839
Detention Length							
<6 months	92	(45.3)	39	(33.9)	53	(60.2)	<0.001
6 - <12 months	56	(27.6)	37	(32.2)	19	(21.6)	0.096
12+ months	55	(27.1)	39	(33.9)	16	(18.2)	0.016
Had poor/fair health pre-detention (Ref.: good/very good/excellent)	82	(40.4)	56	(48.7)	26	(29.5)	0.004
Had health insurance pre-detention	98	(48.3)	62	(53.9)	36	(40.9)	0.062
Has any medical condition -excluding mental illness	164	(80.8)	104	(90.4)	60	(68.2)	<0.001

Note: * Based on two-tailed tests (chi-squared for binary variables; *t*-tests for continuous variables).

### Diagnosed mental illness

115 participants (56.7 %) reported a diagnosed mental illness, most commonly depression (99/115, 86.1 %) and PTSD (65/115, 56.5 %); see **Supplementary Table 1**. There were no statistical differences by MI status in terms of age, gender, ethnicity, educational attainment, criminal record status, or pre-detention access to health insurance (Table 1). However, individuals with MI were more likely to report poor health in the year prior to detention, to experience longer periods of detention (12 months or more), and to have another (non-MI) medical condition, compared to those without MI.

### Outcomes

145 participants (71.4 %) had poor health during detention, 116 (57.1 %) had difficulty accessing medical services, 84 (41.4 %) had difficulty accessing mental health services, 74 (36.5 %) experienced interruptions in care during detention, and 92 (45.3 %) experienced solitary confinement. While the prevalence of these outcome variables was high for all, individuals with MI were more likely to experience them **(**Table 1**)**.

### Multivariable analyses

Table 2 displays odds ratios and goodness of fit statistics from unadjusted and adjusted logistic regression models predicting each outcome. All models show a strong and positive correlation between diagnosed mental illness and each outcome. Referring to the adjusted models, compared to those without MI, participants with MI had much higher odds of poor health (OR 2.80, SE 1.08, *P* *=* *0.01*); difficulty accessing medical services (OR 3.84, SE 1.36, *P* *<* *0.001*) and mental health services (OR 3.17, SE 1.16, *P* *<* *0.001*) while detained; experiencing interruption in care (OR 4.16, SE 1.67, *P* *<* *0.001*); and experiencing solitary confinement (OR 2.44, SE 0.86, *P* *=* *0.01*). With regard to covariates, having poor health in the year prior to detention was associated with increased odds of poor health during detention (OR 4.07, SE 1.77, *P* *<* *0.001*), decreased odds of difficulty accessing medical services while detained (OR 0.46, SE 0.16, *P* = 0.02), and increased odds of experiencing interruption in care (OR 2.04, SE 0.72, *P* = 0.04). Having any other (non-MI) medical condition was associated with increased odds of poor health during detention (OR 3.03, SE 1.37, *P* *=* *0.01*).

**Table 2:** Odds ratios from unadjusted and adjusted models predicting health-related detention experiences.

	Poor Health	Poor Health	Difficulty accessing medical services	Difficulty accessing medical services	Difficulty accessing mental health services	Difficulty accessing mental health services	Care interrupted	Care interrupted	Experienced solitary confinement	Experienced solitary confinement
	b (SE) [p]	b (SE) [p]	b (SE) [p]	b (SE) [p]	b (SE) [p]	b (SE) [p]	b (SE) [p]	b (SE) [p]	b (SE) [p]	b (SE) [p]
Mental Illness	4.49⁎⁎⁎	2.80⁎⁎	3.30⁎⁎⁎	3.84⁎⁎⁎	3.87⁎⁎⁎	3.17⁎⁎	5.77⁎⁎⁎	4.16⁎⁎⁎	2.69⁎⁎⁎	2.44*
	(1.50)	(1.08)	(0.98)	(1.36)	(1.21)	(1.16)	(2.00)	(1.67)	(0.80)	(0.86)
	[0.00]	[0.01]	[0.00]	[0.00]	[0.00]	[0.00]	[0.00]	[0.00]	[0.00]	[0.01]
Age (years) mean (SD)		1.00		1.00		1.00		0.99		1.00
		(0.02)		(0.02)		(0.02)		(0.02)		(0.02)
		[0.93]		[0.84]		[0.84]		[0.58]		[0.81]
Male (Ref.: Female)		0.81		0.89		0.79		1.06		1.22
		(0.54)		(0.44)		(0.37)		(0.56)		(0.58)
		[0.76]		[0.82]		[0.61]		[0.91]		[0.67]
High School Degree or more (Ref.: Less than HS)		0.99		1.39		1.03		0.93		1.91
		(0.40)		(0.49)		(0.35)		(0.35)		(0.67)
		[0.98]		[0.35]		[0.93]		[0.85]		[0.07]
Hispanic/Latina/o (Ref.: Not Latino)		1.65		1.33		0.75		0.39⁎⁎		1.55
		(0.75)		(0.49)		(0.27)		(0.14)		(0.58)
		[0.27]		[0.43]		[0.42]		[0.01]		[0.24]
Has a criminal record (Ref.: No criminal record)		0.62		1.82		0.73		1.34		2.90⁎⁎
		(0.27)		(0.66)		(0.27)		(0.56)		(1.10)
		[0.27]		[0.10]		[0.40]		[0.48]		[0.00]
Detention Length (Ref. <6 months)		2.36		0.75		1.13		0.99		1.36
6 - <12 months		(1.11)		(0.30)		(0.45)		(0.39)		(0.54)
		[0.07]		[0.47]		[0.75]		[0.98]		[0.44]
12+ months		2.05		1.71		2.27*		1.49		2.13*
		(0.89)		(0.67)		(0.87)		(0.63)		(0.81)
		[0.10]		[0.17]		[0.03]		[0.34]		[0.05]
Had poor/fair health pre-detention (Ref.: good/very good/excellent)		4.07⁎⁎		0.46*		0.80		2.04*		1.03
		(1.77)		(0.16)		(0.26)		(0.72)		(0.34)
		[0.00]		[0.02]		[0.50]		[0.04]		[0.94]
Had health insurance pre- detention (Ref.: No ins. pre-det.)		0.84		0.96		1.48		1.48		0.59
		(0.32)		(0.30)		(0.47)		(0.50)		(0.19)
		[0.64]		[0.90]		[0.22]		[0.25]		[0.11]
Has any medical condition -excluding mental illness		3.03*		1.47		1.27		2.50		2.48*
		(1.37)		(0.64)		(0.58)		(1.37)		(1.14)
		[0.01]		[0.38]		[0.60]		[0.09]		[0.05]
Constant	1.20	0.39	0.69	0.26	0.31⁎⁎⁎	0.33	0.19⁎⁎⁎	0.14*	0.47⁎⁎⁎	0.05⁎⁎⁎
	(0.26)	(0.41)	(0.15)	(0.22)	(0.08)	(0.28)	(0.06)	(0.13)	(0.11)	(0.04)
	[0.40]	[0.37]	[0.09]	[0.10]	[0.00]	[0.19]	[0.00]	[0.04]	[0.00]	[0.00]
Observations	203	203	203	203	203	203	203	203	203	203
AIC	225.05	214.8	264.4	267.8	259.1	269.7	240.3	243.1	272.0	268.9
BIC LRchi2-test	231.7	254.5 30.27	271.0	307.6 16.6	265.7	309.5 9.37	246.9	282.9 17.23	278.7	308.7 23.06
		[<0.001]		[0.08]		[0.50]		[0.07]		[0.01]

Table reports odds ratios from multivariable logistic regression with robust standard errors in parentheses, and p-values in brackets; Akaike Information Criterion (AIC) and Bayesian Information Criterion (BIC) provide tests of goodness of fit and parsimony of each model, wherein smaller AIC & BIC values represent better model fit. The Likelihood Ratio (LR) chi2 tests the difference in fit statistics between the fully adjusted model and the base model. A p-value≤0.05 indicates that the fully adjusted model fits better than the base model for any given outcome variable.

⁎ *p* < 0.05,.

⁎⁎ *p* < 0.01,.

⁎⁎⁎ *p* < 0.001.

**Fig. 1:**
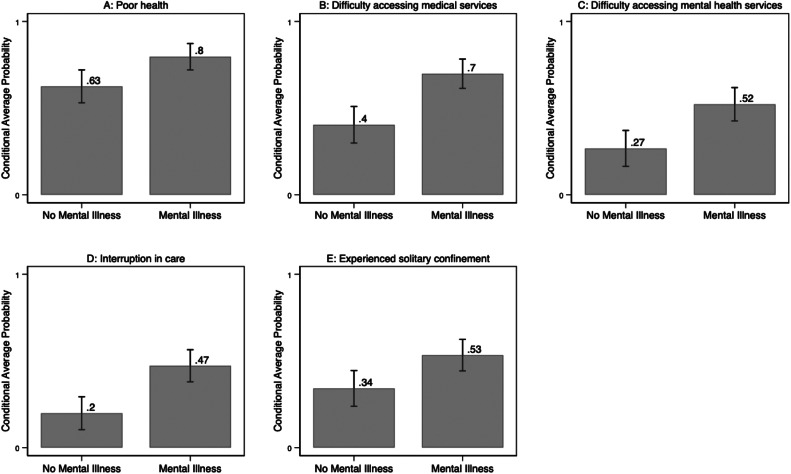
Conditional average probability of health-related detention experiences, by mental illness status.

Fig. 1 displays the conditional average probability of each outcome, by MI status, based on the fully adjusted regression models in Table 2. The conditional average probability of poor health while detained was 0.80 (95 % CI, 0.72–0.87) among those with MI vs. 0.63 (95 % CI, 0.53- 0.72) among those without MI (p-value of difference between MI/no MI=0.01). The conditional average probability of difficulty accessing medical services while in immigration detention was 0.70 (95 % CI, 0.62–0.78) among those with MI, compared to 0.40 (95 % CI, 0.30–0.51) among those without MI (p-value of difference <0.001). The conditional average probability of difficulty accessing mental health services was 0.52 (95 % CI, 0.43–0.62) among those with MI, compared to 0.27 (95 % CI, 0.17–0.37) among those without MI (p-value of difference<0.001). The conditional average probability of care interruption was 0.47 (95 % CI, 0.38–0.56) for those with MI, compared to 0.20 (95 % CI 0.11–0.29) for those without MI (p-value of difference<0.001) Finally, the conditional average probability of experiencing solitary confinement was 0.53 (95 % CI, 0.44–0.62) for those with MI vs. 0.34 (95 % CI, 0.24–0.44) for those without MI (p=value of difference=0.01).

## Discussion

This study generates three main findings. First, over half of our study participants (56.7 %; 115/203) reported a diagnosed MI, with depression and PTSD being most common. Second, there was a high prevalence of poor health, difficulty accessing care, care interruptions, and experience of solitary confinement across all participants, but especially among those with MI. Third, participants with MI had approximately three to five times higher odds of poor health, greater difficulty accessing medical and mental health services, respectively, greater likelihood of interruptions to care while detained, and greater likelihood of experiencing solitary confinement, relative to those without MI. These results are robust to the inclusion of covariates.

The higher prevalence of MI among participants in our sample of formerly detained immigrants (56.7 %) is consistent with the disproportionate number of individuals with MI in the imprisoned population in the United States: 64 % in local jails, 56 % in state prisons, and 45 % in federal prisons (James and Glaze, 2016). Our results are also similar to a previous study of 71 detained asylum seekers in the US, the majority of whom had clinically significant symptoms of PTSD (50 %), anxiety (77 %), or depression (86 %) (Keller et al., 2003). The prevalence in our sample is higher than in other studies of detained and formerly detained immigrants, where it ranged from 17 to 19 % (Bakely et al., 2023; Patler and Saadi, 2021), as well as in the non-institutionalized US population where about 26 % of adults suffer from a diagnosable mental disorder (National Institute of Mental Health (NIMH) 2022), The higher prevalence of MI in our and other samples of detained immigrants may be related to a series of social-structural determinants of health that span pre-migration, migration, and settlement experiences even prior to detention (e.g., lack of access to legal entry to the United States, stress due to discrimination and/or legal status vulnerability, and more) (Saadi et al., 2020). Structural features of the US immigration detention system can also cause and/or worsen MI through various mechanisms including severing of social and family connections and health-harming conditions of confinement such as overcrowding, unsanitary conditions, sleep deprivation, verbal and/or physical abuse, lack of recreation time, and exposure to extreme temperatures or weather conditions (Bakely et al., 2023; Diaz et al., 2023; Saadi et al., 2022; Disability Rights California 2019).

Our second and third sets of results reveal a high prevalence of poor health status, difficulties accessing medical and mental health care, and care interruptions among all detained immigrants in our sample, but especially among those with MI. In addition to harmful conditions of confinement, US immigration detention facilities often fail to provide adequate medical and mental health services and preventative care, in some cases to the point of medical negligence, which can place detained people—perhaps especially those with MI—at risk for worsening health or even death (Nwadiuko et al., 2022; Venters and Keller, 2009; Dekker et al., 2024; Kelly, 2018; Grassini et al., 2021).

We also found that nearly one in three participants without MI, and more than half of participants with MI, experienced solitary confinement during their time in ICE custody. This is comparable to the population prevalence of solitary confinement in the criminal legal system (Beck, 2015; Pullen-Blasnik et al., 2021). The United Nations argues solitary confinement should never last longer than 15 days for anyone, and should be banned altogether for individuals with MI, as it can uniquely harm their health, in violation of international human rights standards (United Nations 2011; United Nations 2016). Moreover, immigration detention staff are often inadequately trained to handle psychiatric healthcare, and often use solitary confinement to manage psychiatric emergencies (Project on Government Oversight 2019). Our results provide evidence that solitary confinement is regularly imposed upon detained immigrants in general, and especially those with MI, despite its unique harms to their health (Franco et al., 2022; Patler et al., 2018).

Studies of the health of immigrants detained in the United States are rare, in large part due to systemic barriers accessing reliable data on detained immigrants, an issue we discuss further below. Our study, facilitated through a research-practitioner partnership with a national civil rights organization, provides evidence that the US immigration detention is linked to high rates of poor health, difficulty accessing care, care interruptions, and punitive experiences like solitary confinement, especially among individuals with MI, who experience unique vulnerabilities that can place them at increased risk of harm. Our results therefore make a valuable contribution to research linking detention to poor or worsened physical and mental health outcomes, which is currently based largely on the experiences of refugees or asylum seekers outside the United States (von Werthern et al., 2018; Steel et al., 2006; Essex et al., 2022).

### Limitations and opportunities for future research

This research generates important questions for future study. First, analyses of representative data on detained immigrants are essential, yet ICE discloses very limited demographic information about the populations it detains (e.g., aggregate annual statistics on country of origin and criminality), making it impossible to calculate even basic population-level estimates, let alone analyze health statistics. Detained immigrants are also the only cohort of patients with federally sponsored healthcare for which federal health data is not released. Further, detention under US immigration law is not a sentence with a formal end date, and there is no systematic mechanism for release from detention (e.g., bond) that could allow researchers to systematically sample currently or recently detained immigrants (Patler and Golash-Boza, 2017). Convenience or referral samples of current or formerly detained immigrants, such as the present study, are therefore one of few ways to directly assess the health of this population (von Werthern et al., 2018; Patler and Branic, 2017; Patler et al., 2021; Diaz et al., 2023). Researchers should continue to strive to acquire data that would allow for representative analyses, while ensuring privacy, safety, and confidentiality for detained people.

Given the systemic barriers to accessing representative data on detained immigrants, our study's goal was to descriptively examine the experiences of recently detained immigrants with and without MI. Still, our results should be interpreted with some caveats. For example, it is possible that release of the individuals in our sample during the COVID-19 pandemic may have included people with higher rates of chronic conditions, including mental illness (Patler and Saadi, 2021) Assessing this possibility is not possible given the dearth of health data on detained immigrants. However, we attempted to adjust for this within our sample by including several pre-detention health measures as covariates in our model. Relatedly, if stricter or more dangerous detention during the COVID-19 pandemic caused increased distress leading to higher rates of MI diagnoses, or if release during the pandemic was more common among medically vulnerable people, this could influence the prevalence of MI and/or self-reported poor health in our sample relative to detained immigrants before or after the pandemic (Williams et al., 2024). Future work should seek to parse out the potential distinctiveness of the pandemic period.

Finally, the relationship between mental health and detention is complex and there is likely a bidirectional relationship between detention and mental health (Diaz et al., 2023; Gerlach, 2022; Massoglia and Pridemore, 2015). While incarceration may cause or exacerbate mental illness (Schnittker et al., 2012), people with mental illness also face heightened vulnerability to health-related harms while incarcerated. Studies find increased physical and sexual victimization (Blitz et al., 2008; Crisanti and Frueh, 2011), risk of self-harm and suicide, and exposure to solitary confinement (Patler et al., 2018) among incarcerated individuals. It is also possible that those with mental illness have poorer health even prior to detention which may lead to increased risk of poor outcomes while detained, regardless of MI status. To account for the possible relationship between pre-detention health and poor outcomes while detained, our models control for poor health and access to health insurance prior to detention, and lifetime diagnosis of any other (non-MI) medical condition. Even with the inclusion of these covariates, the association between MI and our health outcomes persists (Table 2 and Fig. 1).

Our sample size also impedes further disaggregation (e.g., by racialized group, gender identity, language background, etc.) and therefore may not capture important differences within and between groups of detained people. We encourage future work that can underscore the experiences of multiply marginalized detained immigrants, such as immigrants racialized as Black, who experience disproportionate rates of detention and solitary confinement, as well as other vulnerable groups such as Indigenous migrants, women, unaccompanied minors, and transgender immigrants (Franco et al., 2022; Boehm and Terrio, 2019; Menjívar et al., 2021; Vogler and Rosales, 2023; Doubossarskaia et al., 2024)

These limitations notwithstanding, as one of very few studies of detained immigrants' health in the United States, our results provide an important foundation for understanding the health-related detention experiences of detained immigrants with and without mental illness (Plevin and R, 2020; Kelly, 2018; Fadel and Dreisbach, 2023; OIG 2017). We document significant gaps in health-related detention outcomes between individuals with and without MI. These gaps, and the mechanisms producing them, should be urgently addressed.

### Conclusion

This study has important implications for reducing health harms in immigrant communities. Policy changes can mitigate the health harms of immigration detention by limiting the use of detention for vulnerable individuals, including those with mental illness, and ensuring consistent and thorough identification and monitoring. Although related ICE directives exist, medical experts have determined they lack meaningful oversight, are underenforced, and are likely inadequate even if fully enforced (National Immigrant Justice Center 2022; U.S. Immigration and Customs Enforcement 2022). Additional strategies could target the unique needs and vulnerabilities of those with MI by barring the use of solitary confinement and expanding access to counsel in immigration proceedings.

The most effective harm-reduction policy would be to end the use of incarceration in immigration legal proceedings. US immigration laws are civil, not criminal; thus, detained immigrants are not serving a sentence, but being held administratively as their deportation cases proceeds. The US government argues that detention is necessary to ensure court appearances. However, a comprehensive study of all removal proceedings from 2008 to 2018 found only small differences in court appearance rates between individuals with and without a history of detention (Eagly and Shafer, 2020). Further, at least two studies have demonstrated health improvements following release from US immigration detention (Keller et al., 2003; Patler et al., 2021). Ending detention could therefore mitigate some of the health harms immigrants experience, especially those associated with unique experiences of detention such as solitary confinement, without substantively influencing immigration legal proceedings (Patler et al., 2023; Lopez et al., 2021). All of this must be done within a larger call for oversight and transparency in immigration detention, to improve health outcomes and equity for detained immigrants (Dekker et al., 2024).

## CRediT authorship contribution statement

**Caitlin Patler:** Writing – review & editing, Writing – original draft, Supervision, Project administration, Methodology, Investigation, Funding acquisition, Formal analysis, Data curation, Conceptualization. **Altaf Saadi:** Writing – review & editing, Methodology, Funding acquisition, Conceptualization. **Paola Langer:** Writing – review & editing, Formal analysis.

## Declaration of competing interest

The authors declare that they have no known competing financial interests or personal relationships that could have appeared to influence the work reported in this paper.

## Appendix Supplementary materials

Image, application 1

## Footnotes

1 Some, but not all, of the cases prioritized the release of individuals with medical conditions that may have placed them at higher risk for poor outcomes from COVID-19; see limitations section.
