# Supplementary material for: The health-related experiences of detained immigrants with and without mental illness
Source: J Migr Health. 2025 Jan 4;11:100302. doi: 10.1016/j.jmh.2025.100302 (PMC11773275; doi:10.1016/j.jmh.2025.100302)
Supplement: Supplementary file 1 [file mmc1.docx]

**Appendix**

**Supplementary Table 1. Distribution of mental health conditions among those with a diagnosed mental illness**

|  | *n (%)* |  |  |
| --- | --- | --- | --- |
| **Variable** |  |  |  |
| Depression | 99 | (86.1) |  |
| PTSD | 65 | (56.5) |  |
| Schizophrenia or Bipolar Disorder | 18 | (15.7) |  |
| Other mental health problem/condition | 36 | (31.3) |  |
|  | | |  |

Note: 32% of the total sample (n=66/203), or 57% of those with DMI, has two or more mental illness diagnoses.
